# Primary care intervention to address cardiovascular disease medication health literacy among Indigenous peoples: Canadian results of a pre-post-design study

**DOI:** 10.17269/s41997-018-0034-9

**Published:** 2018-03-09

**Authors:** Janet Smylie, Kristen O’Brien, Chloé G. Xavier, Marcia Anderson, Constance McKnight, Bernice Downey, Margaret Kelaher, De dwa da dehs nye>s Aboriginal Health Centre

**Affiliations:** 1 Well Living House, Centre for Urban Health Solutions (CUHS) in the Li Ka Shing Knowledge Institute, St. Michael’s Hospital, 30 Bond Street, Toronto, ON Canada; 2 Dalla Lana School of Public Health, University of Toronto, Toronto, ON Canada; 3 Ongomiizwin Indigenous Institute of Health and Healing, Rady Faculty of Health Sciences, University of Manitoba, Winnipeg, MB Canada; 4 De dwa da dehs nye>s Aboriginal Health Centre, Hamilton, ON Canada; 5 Faculty of Health Sciences, McMaster University, Hamilton, ON Canada; 6 Centre for Health Policy, Melbourne School of Population and Global Health, University of Melbourne, Melbourne, Australia

**Keywords:** Indigenous, Cardiovascular disease, Health literacy, Indians, North American, Autochtone, maladies cardiovasculaires, compétence informationnelle en santé, Indiens d’Amérique Nord

## Abstract

**Context:**

Cardiovascular diseases (CVD) are a leading cause of illness and death for Indigenous people in Canada and globally. Appropriate medication can significantly improve health outcomes for persons diagnosed with CVD or for those at high risk of CVD. Poor health literacy has been identified as a major barrier that interferes with client understanding and taking of CVD medication. Strengthening health literacy within health services is particularly relevant in Indigenous contexts, where there are systemic barriers to accessing literacy skills.

**Objective:**

The aim of this study is to test the effect of a customized, structured health literacy educational program addressing CVD medications.

**Methods:**

Pre-post-design involves health providers and Indigenous clients at the De dwa da dehs nye>s Aboriginal Health Centre (DAHC) in Ontario, Canada. Forty-seven Indigenous clients with or at high risk of CVD received three educational sessions delivered by a trained Indigenous nurse over a 4- to 7-week period. A tablet application, pill card and booklet supported the sessions. Primary outcomes were knowledge of CVD medications and health literacy practices, which were assessed before and after the programe.

**Results:**

Following the program compared to before, mean medication knowledge scores were 3.3 to 6.1 times higher for the four included CVD medications. Participants were also more likely to refer to the customized pill card and booklet for information and answer questions from others regarding CVD.

**Conclusions:**

This customized education program was highly effective in increasing medication knowledge and health literacy practice among Indigenous people with CVD or at risk of CVD attending the program at an urban Indigenous health centre.

## Résumé

**Contexte:**

Les maladies cardiovasculaires (MCV) sont l’une des principales causes de morbidité et de mortalité chez les Autochtones au Canada et ailleurs dans le monde. Des médicaments appropriés peuvent sensiblement améliorer les résultats sanitaires des personnes ayant un diagnostic de MCV ou de celles qui présentent un risque élevé de MCV. Une maîtrise déficiente de l’information en matière de santé serait un obstacle majeur à la compréhension des usagers et à la prise de médicaments pour traiter les MCV. Le renforcement de la maîtrise de l’information en santé au sein des services de santé est particulièrement pertinent en milieu autochtone, où il existe des barrières systémiques à l’acquisition de capacités de lecture et d’écriture.

**Objectif:**

Tester l’effet d’un programme personnalisé et structuré de maîtrise de l’information en santé axé sur les médicaments pour traiter les MCV.

**Méthode:**

Notre plan d’étude avant/après a impliqué des dispensateurs de soins de santé et des usagers autochtones du centre de santé autochtone De dwa da dehs nye>s en Ontario, au Canada. Quarante-sept usagers autochtones atteints de MCV ou à risque élevé de contracter des MCV ont assisté sur une période de quatre à sept semaines à trois séances éducatives données par une infirmière autochtone formée. Une application pour tablette, un distributeur de pilules et un livret venaient avec les séances. Les principaux résultats étudiés étaient la connaissance des médicaments pour traiter les MCV et la maîtrise de l’information en santé; ils ont été évalués avant et après le programme.

**Résultats:**

Après le programme, les notes moyennes obtenues pour la connaissance des médicaments étaient de 3,3 à 6,1 fois plus élevées pour les quatre médicaments inclus dans l’étude. Les participants étaient aussi plus susceptibles de consulter leur distributeur de pilules personnalisé et leur livret pour y trouver de l’information, et de répondre aux questions d’autres personnes au sujet des MCV.

**Conclusions:**

Ce programme éducatif personnalisé mené dans un centre de santé autochtone en milieu urbain a très efficacement accru la connaissance des médicaments et la maîtrise de l’information en santé chez les Autochtones atteints de MCV ou à risque de contracter des MCV qui l’ont suivi.

## Introduction

Deaths and hospitalizations linked to cardiovascular diseases (CVD) such as stroke and heart disease are second only to cancer as a cause of death in Canada (Statistics Canada 2011). As a result of improvements in the treatment of risk factors such as elevated cholesterol and blood pressure and better medical and surgical treatments, rates have recently been declining for the general Canadian population (Smith and The 2009; Wijeysundera et al. 2010; Statistics Canada n.d.-a). In contrast, Indigenous people in Canada (First Nations, Métis and Inuit) experience a disproportionate burden of CVD (Reading 2015; Atzema et al. 2015) and do not appear to be experiencing the same CVD rate declines. While Indigenous health surveillance systems in Canada are patchy (Smylie and Firestone 2015), the data we do have are convincing. For example, for First Nations populations, all-cause circulatory disease mortality rates actually increased for First Nations women between the 1980s and the 2000s. For First Nations men during the same time period, mortality rate decreases were much smaller than for Canadian men (Young and Cardiovascular 2012). As a result, First Nations mortality rates from circulatory disease are now higher than those of non-First Nations comparison groups (Young and Cardiovascular 2012; Health Canada 2014; Tjepkema et al. 2012). Multiple studies have also documented higher population-based prevalence rates of heart disease and linked risk factors, including diabetes, hypertension, obesity, and commercial tobacco use, for Indigenous compared to non-Indigenous populations in Canada in both urban and rural/remote regions (Young and Cardiovascular 2012; First Nations Information Governance Centre (FNIGC) 2012; Smylie et al. 2011; Firestone et al. 2014; Chow et al. 2005).

These striking Indigenous/non-Indigenous CVD inequities are regrettably predictable given the known disparities in the determinants of CVD health, including healthy diet, regular exercise, commercial tobacco smoking cessation, treatment of medical risk factors, and appropriate medical and surgical management post-diagnosis. The unequal burden of poverty and food insecurity experienced by Indigenous peoples in Canada has been well documented. The roots of these roots can be found in historic and current governmental policies that disrupt Indigenous societies, economies, and food supplies, including the illegal appropriation of and resource extraction from Indigenous lands, forced community relocations, residential schools and the Sixties Scoop (Truth and Reconciliation Commission of Canada 2015). Smoking rates for Indigenous people in Canada are approximately twice the rate of the non-Indigenous population (Statistics Canada n.d.-b), and the significant decreases in tobacco use among the general Canadian population have not been shared in Indigenous communities. Indigenous peoples experience inequities in timely access to life-saving acute treatments for CVD globally and domestically (Bresee et al. 2014; Canadian Institute of Health Information 2013; Lopez et al. 2014; Williams 2007). For example, Bresee and colleagues (Bresee et al. 2014) found that First Nations persons across urban, rural and remote geographies were less likely to receive coronary angiography within 24 h of a myocardial infarction (OR 0.73) and more likely to die (OR 1.30) compared to the non-First Nations population.

Reduction of total cholesterol and systolic blood pressure through appropriate use of CVD medications is known to reduce CVD mortality (Wijeysundera et al. 2010; Baigent et al. 2005; Wang et al. 2005). Policy and practice responses to these findings have included significant investments in ensuring clinicians are optimizing pharmaceutical and lifestyle management of these conditions using clinical practice guidelines. The premise appears to be that if health care providers are prescribing the correct medications, outcomes will improve. There is a gap in research specifically examining the degree to which health systems and health care providers are facilitating equitable patient access to the information they need to be able to take their CVD medications correctly.

The Canadian Expert Panel on Health has defined health literacy as “the ability to access, understand, evaluate and communicate information as a way to promote, maintain and improve health in a variety of settings across the life-course (p.11)” (Rootman and Gordon-El-Bihbety 2008). Recognized as a critical element for addressing and designing effective health services, health literacy is also considered a social determinant of health and a driver of ethnic health status disparities. For example, the Institute of Medicine (Institute of Medicine (US) Committee on Health et al. 2004) estimates that health literacy accounts for 25–30% of ethnic differences in health outcomes. For Indigenous peoples in Canada, health literacy opportunities and challenges are interwoven with colonial policies such as the historic outlawing of our languages and emerging approaches in Indigenous education (Smylie et al. 2006).

As part of our pre-trial investigations, our research team identified that among a sample of Indigenous patients with CVD from our proposed study site, the majority had International Assessment of Literacy Skills (IALS) scores that were lower than Canadian averages and would make correct use of CVD medications difficult (Downey et al. 2013). This is unsurprising given the known educational inequities experienced by Indigenous people in Canada (Neeganagwedgin 2011). We also investigated health care provider (HCP) understandings of health literacy in Canada and internationally (Downey et al. 2013; Lambert et al. 2014). We found that baseline HCP conceptualizations of health literacy were primarily focused on patient capacities, with some recognition of deficits in system-level supports, including limited time and the need for training and accessible information (Downey et al. 2013). Overall, there was a clear opportunity to develop and implement a system-level health service intervention to support HCPs and their Indigenous patients to enhance their health literacy skills and practices and also to reduce the commonly unrecognized demands placed on Indigenous patients (Lambert et al. 2014).

This paper reports on the Canadian results of a multi-site international study involving Australia, Canada and New Zealand. The primary objective of this Canadian study was to work in partnership with an Indigenous health service to test the effect of a customized, structured health literacy educational program addressing CVD medications.

## Methods

### Setting and participants

This study was conducted in partnership with the De dwa da dehs nye>s Health Access Centre (DHAC), a comprehensive Indigenous health service which provides primary health care, traditional healing and health promotion programs to Indigenous peoples living in the cities of Hamilton and Brantford, ON, Canada. These two cities are located approximately 40 km apart in southern Ontario on the traditional territories of Haudenosaunee (Iroquoian) and Anishnawbe (Ojibway) peoples. There are two nearby First Nations, Six Nations of the Grand River and Mississaugas of the New Credit. According to the National Household Survey, in 2011, the Indigenous population in the city of Hamilton by ancestry was 15,840, comprising 3.1% of the total population (509,635), and the Indigenous population in the city of Brantford by ancestry was 5440, comprising 5.9% of the total population (91,975) (Statistics Canada 2013). Our research group has recently demonstrated elsewhere that 2011 NHS estimates of Indigenous peoples in southern Ontario cities underestimate the actual population size by a factor of 2–4, so these figures need to be interpreted accordingly.

Indigenous clients of the health service were eligible for the study if they could provide informed consent, were aged 20 years or older, had a history of at least one CVD event (angina pectoris, myocardial infarction, transient ischaemic attack or stroke) or had ≥ 15% risk of CVD over the next 5 years, and were prescribed at least two of the following medication classes: statin, aspirin, ACE inhibitors or beta blockers. Potential participants were identified through a customized search of the clinic electronic medical record system for target study medications and referral from primary health care providers. Clients indicating an interest in volunteer study participation to health service staff not directly involved in the trial were contacted by the study nurse. Family members of participants were invited to sit in on study sessions.

### Design

The intervention design is a single arm pre-post trial with multiple measurement points. Factors precluding the use of a randomized, controlled design have been detailed elsewhere (protocol paper) and include the relatively small eligible study population and a high likelihood of contamination between intervention and control groups who are all clients of the DHAC and may also be otherwise networked through the local Indigenous community.

In keeping with benchmark ethical requirements and evidence-based practice in Indigenous health research (Smylie et al. 2012; Leadbeater et al. 2011), the DHAC was comprehensively involved in the proposal development, research study design and implementation, and results analysis, documentation and dissemination. We applied an Indigenous community participatory action research partnership method that had been successfully demonstrated in a previous community-partnered, community-implemented health needs assessment project (Smylie et al. 2011; Firestone et al. 2014). The research team was led by Indigenous people, including the executive director of DHAC who was a project co-investigator and the study nurse who was a DHAC employee. There was a local project research committee comprised of DHAC staff. Indigenous community governance and management of research data and publications was formalized through a signed research, data-sharing and publication agreement. In addition, the study was approved by the St. Michael’s Hospital Research Ethics Board.

This Canadian study is part of a larger international trial involving three additional Indigenous health services in Australia and New Zealand. Pooled study results are to be presented elsewhere. This larger study is registered with the Australian and New Zealand Clinical Trials Registry (ACTRN12612001309875; date of registration 18/12/2012).

### Intervention

**Fig. 1:**
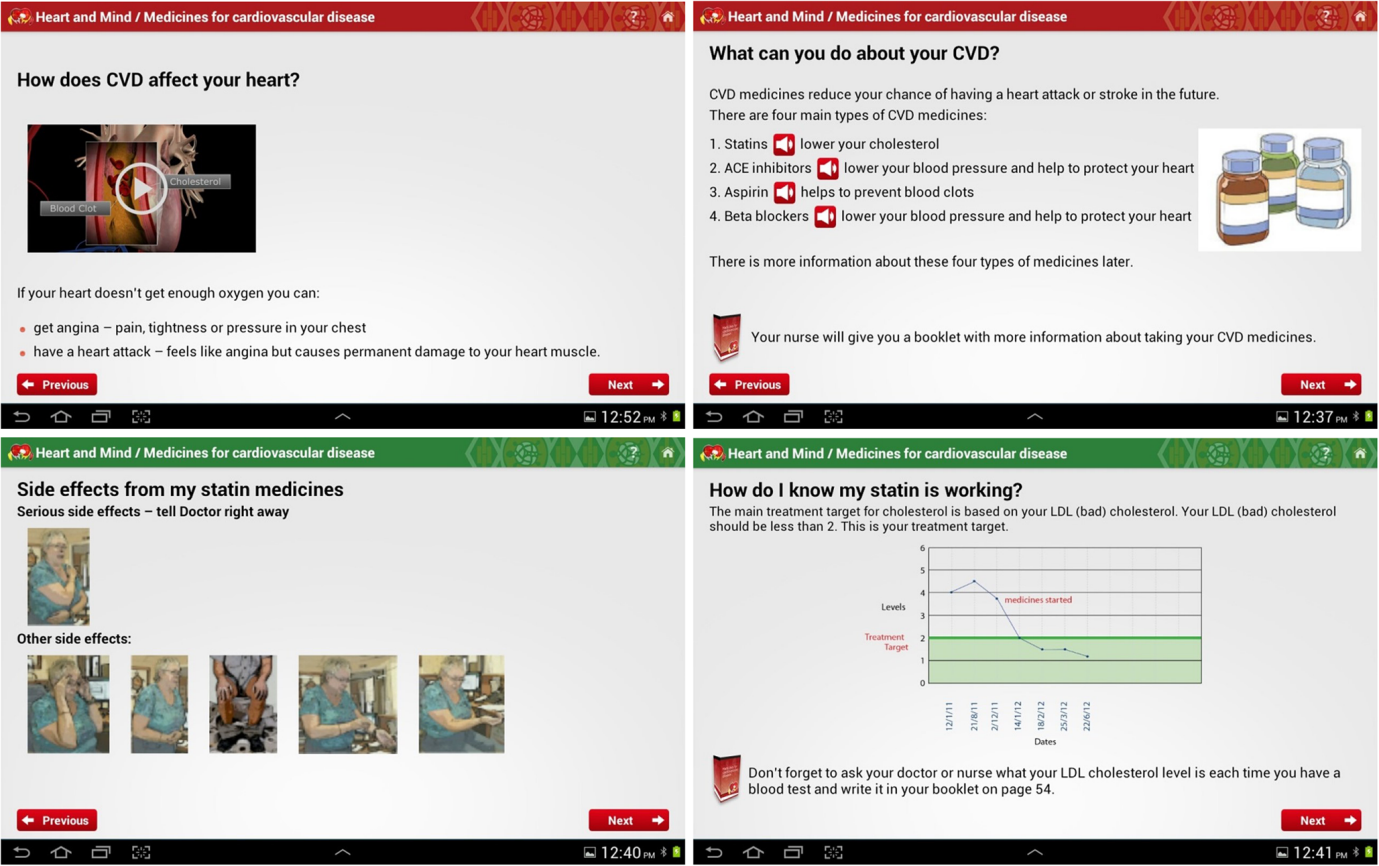
Screen shots from interactive tablet application

**Fig. 2:**
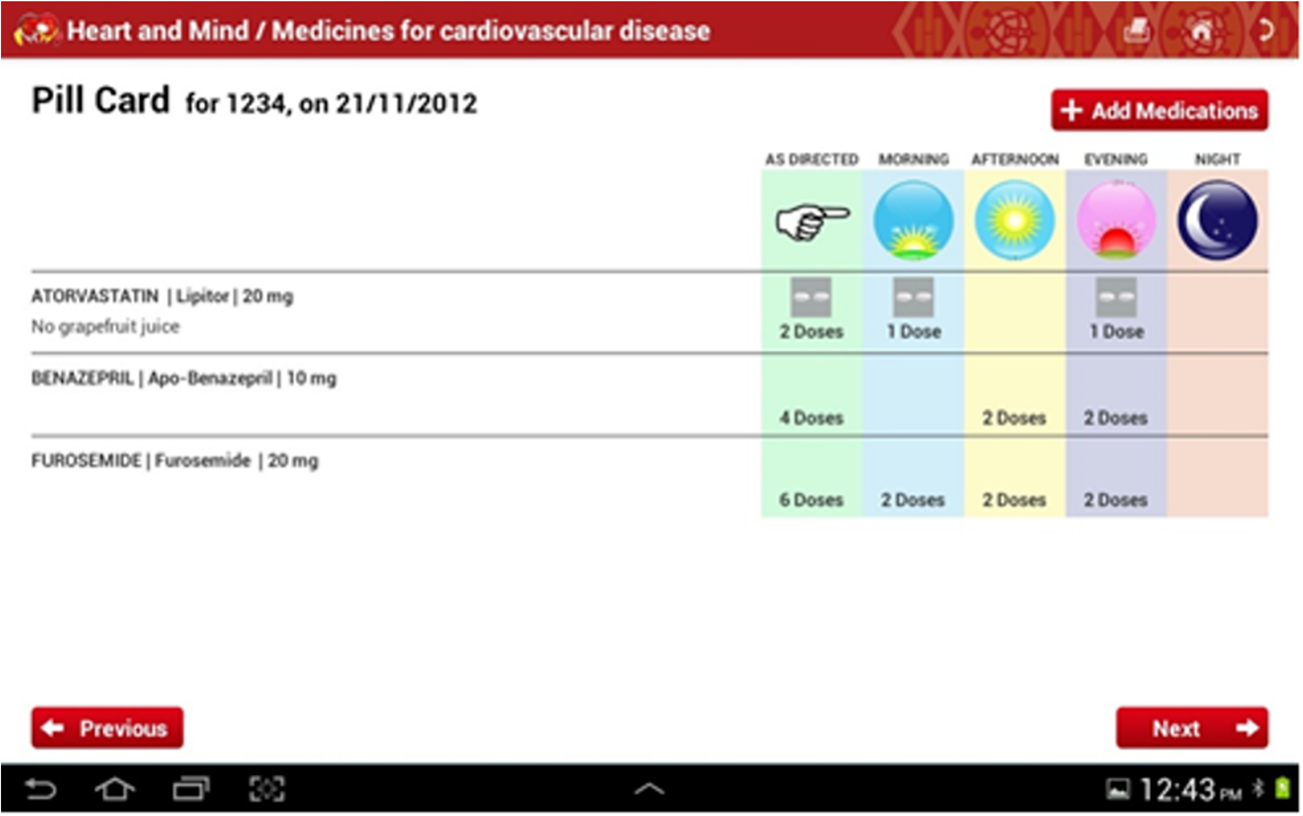
Sample custom pill card

The intervention is described in detail elsewhere (Crengle et al. 2014). Briefly, it consisted of three sequential educational sessions with an Indigenous nurse who had received specific training in health literacy, including Indigenous adult literacy strategies that support the uptake of knowledge and health literacy skills. Educational sessions were scheduled to optimize learning, with the second session planned for 7 days after the first session (with a 1-week grace period) and the third session planned for 28 days after the first session (with a 4-week grace period). At the first session, participants received a customized information booklet designed by the medical and health literacy research team members that was used along with an interactive tablet application to support the educational sessions. The booklet (www.welllivinghouse.com/IHL) contains information about CVD, medication use and the four CVD study medication classes, including scientific and brand names, what the medication does, how to take it, interactions, side effects, contraindications, and lipid and blood pressure targets. The interactive tablet application (Fig. 1) ensured that the nurse covered the CVD medication information in a structured and consistent way and provided interactive opportunities for the participant, including two animations of the pathology that occurs in heart disease and stroke, respectively. The application also produced a participant-specific pill card (Fig. 2). During educational sessions, two and three participants were encouraged by the teaching nurse to come up with questions about their CVD or CVD medications that they would like to ask their doctor or nurse.

### Measures

Participant knowledge of CVD medications and their use, including treatment targets, was collected at baseline (T1) and before and after each of the three educational sessions (see Table 1 for summary of study data collection points). The primary study outcome was change in mean participant knowledge of CVD medications between T1 (pre-intervention baseline) and post-educational sessions 1, 2 and 3 for each of the study CVD medication classes (T2, T4 and T6, respectively). Secondary study outcomes focused on changes in health literacy practices by examining: (a) whether or not participants were more likely to read from their pill bottle, pill care, information booklet or own notes after the intervention compared to baseline; (b) changes in the resources used by patients to get information on their CVD post-intervention compared to pre-intervention; (c) whether or not participants had asked questions of their doctor or nurse; and (d) whether or not participants had answered questions for others in their family or community about CVD and CVD medications.

**Table 1:** Summary of study data collection points

Time point		Measurement
Enrollment	T0	Consent, enrollment and baseline demographic and clinical information
Session 1	T1	Pre-session 1 medication knowledge and health literacy practices
	T2	Post-session 1 medication knowledge and health literacy practices
Session 2 (7 days after session 1)	T3	Pre-session 2 medication knowledge and health literacy practices Health literacy practices between sessions 1 and 2
	T4	Post-session 2 medication knowledge and health literacy practices
Session 3 (28 days after session 2)	T5	Pre-session 3 medication knowledge and health literacy practices Health literacy practices between sessions 2 and 3
	T6	Post-session 3 medication knowledge and health literacy practices

Baseline demographic, medical history (including CVD history or CVD risk), medication information, blood pressure and lipid measures were collected at the enrollment session or before the start of the first educational session. Instruments consisting of 9 to 13 class-specific questions to assess knowledge of the four classes of CVD medications and their use were custom developed, integrated into the tablet application, and completed immediately before and after each of the educational sessions for a total of six medication knowledge measurement points.

Participant’s health literacy practices were assessed by the intervention nurse who had been trained to observe and document on the tablet application which supplemental materials were being used (i.e., pill bottle, customized pill card, intervention booklet or own information) during each educational session and whether or not the client was using this information spontaneously or with prompting.

### Analyses

Data from the tablet applications were securely transmitted to a server and extracted in the form of an Excel spreadsheet. Analyses were conducted using SPSS Statistics (version 22). Descriptive statistics such as means and standard deviations were calculated for continuous measures, while counts and percentages were calculated for discrete measures. Participant characteristics included age, gender, baseline CVD disease information (type of diagnosis, number of diagnoses, time from diagnosis), co-morbidities, baseline CVD medication information, and baseline BP and lipid levels for the Brantford and Hamilton sites, with significance testing for difference across these two sites. Mean drug knowledge scores were then calculated for each of the four CVD drug classes at each measurement point, with significance testing for difference between baseline and post-sessions 1, 2 and 3 applying paired *t* testing. Finally, generalized estimating equation multivariable models with the natural logarithm of medication knowledge score as the dependent (Y) variable were constructed to test whether there was a relative change in medication knowledge score before and after each educational session while controlling for site and baseline diabetes diagnosis.

With respect to health literacy practices, chi-square tests were used to test the association between health literacy practices prior to T1 (before the first education session) and after T6 (end of the third session). We tested for significant differences with respect to participants reading from different sources (pill bottle, pill card, book or notes) and all of these sources during education sessions with prompting and spontaneously. Furthermore, we tested to see if there was an association between the number of participants who reported asking and answering questions at T4 and T6 (post-sessions 2 and 3).

## Results

### Recruitment

We were able to generate a list of 204 potential participants using medications listed in DHAC EMR. Health centre staff were able to contact just under half of these clients by telephone or letter and invite them to participate in the study. The large majority of those contacted indicated to health centre staff that they were interested in learning more about the study and were subsequently contacted by the study nurse for review of eligibility and informed consent. Additional participants were referred to the study nurse directly by their primary care providers. Upon final review of eligibility by the study nurse, 49 participants (27 in Brantford and 22 in Hamilton) were eligible and enrolled in the study. Two participants dropped out of the study before completing all three educational sessions, one from each site. A total of 47 Indigenous participants completed the study and were included in subsequent data analyses, 26 in Brantford and 21 in Hamilton.

### Participant characteristics

Table 2 provides baseline characteristics of participants including age, gender and information on CVD diagnosis, CVD medications, co-morbidities, blood pressure and lipid levels. There were no significant differences in patient characteristics between the Hamilton and Brantford sites. Mean participant age was 58.7 [SD 8.6]. Over two thirds of participants (70.2%) had a diagnosis of CVD prior to entering the study, and the remainder met the inclusion criteria of > 15% risk of CVD over 5 years. For those with pre-existing CVD, the average time since diagnosis was 9.3 years [SD 5.7] and the most common diagnoses were angina (42.6%) and MI (31.9%) followed by TIA (17%) and stroke (4.3%). Co-morbid diabetes (76.6%) and COPD (46.8%) were common.

**Table 2:** Participant characteristics at baseline (T0) by site

	Canada	Brantford	Hamilton	*p* value*
Number of participants, *n* (%)	47 (100)	26 (55.3)	21 (44.7)	
Age				
Years, mean (SD)	58.7 (8.6)	59.4 (9.7)	57.9 (7.1)	0.573
Gender				
Male, *n* (%)	22 (46.8)	11 (42.3)	11 (52.4)	0.491
CVD diagnoses				
Angina, *n* (%)	20 (42.6)	10 (38.5)	10 (47.6)	0.528
MI, *n* (%)	15 (31.9)	8 (30.8)	7 (33.3)	0.851
Stroke, *n* (%)	2 (4.3)	1 (3.8)	1 (4.8)	0.877
TIA, *n* (%)	8 (17.0)	4 (15.4)	4 (19.0)	0.740
Number of CVD diagnosis				
Nil, *n* (%)	14 (29.8)	8 (30.8)	6 (28.6)	0.716
One, *n* (%)	23 (48.9)	14 (53.8)	9 (42.9)	
Two, *n* (%)	8 (17.0)	3 (11.5)	5 (23.8)	
Three or more, *n* (%)	2 (4.3)	1 (3.8)	1 (4.8)	
Time with CVD				
Years, mean (SD)	9.3 (5.7)	10.4 (6.3)	7.9 (4.6)	0.213
Co-morbidity				
Diabetes, *n* (%)	36 (76.6)	18 (69.2)	18 (85.7)	0.185
CHF, *n* (%)	3 (6.4)	1 (3.8)	2 (9.5)	0.429
COPD asthma, *n* (%)	22 (46.8)	14 (53.8)	8 (38.1)	0.282
Gout, *n* (%)	6 (12.8)	2 (7.7)	4 (19.0)	0.181
Peptic ulcer, *n* (%)	7 (14.9)	4 (15.4)	3 (14.3)	0.316
Number of co-morbidities				
None, *n* (%)	7 (14.9)	5 (19.2)	2 (9.5)	0.619
One, *n* (%)	16 (34.0)	8 (30.8)	8 (38.1)	
Two, *n* (%)	17 (36.2)	10 (38.5)	7 (33.3)	
Three, *n* (%)	4 (8.5)	1 (3.8)	3 (14.3)	
Four, *n* (%)	3 (6.4)	2 (7.7)	1 (4.8)	
Five, *n* (%)	0 (0)	0 (0)	0 (0)	
CVD medications at baseline				
Statin, *n* (%)	43 (91.5)	24 (92.3)	19 (90.5)	0.823
ACE, *n* (%)	29 (61.7)	17 (65.4)	12 (57.1)	0.563
BB, *n* (%)	21 (44.7)	12 (46.2)	9 (42.9)	0.821
Aspirin, *n* (%)	35 (74.5)	20 (76.9)	15 (71.4)	0.668
Number of medications				
Two, *n* (%)	21 (44.7)	10 (38.5)	11 (52.4)	0.611
Three, *n* (%)	19 (40.4)	12 (46.2)	7 (33.3)	
Four, *n* (%)	7 (14.9)	4 (15.4)	3 (14.3)	
Allergy/side effect				
Statin, *n* (%)	3 (6.4)	1 (3.8)	2 (9.5)	0.429
ACE inhibitor, *n* (%)	1 (2.1)	0 (0)	1 (4.8)	0.261
BB, *n* (%)	0 (0)	0 (0)	0 (0)	NA
Aspirin, *n* (%)	3 (6.4)	1 (3.8)	2 (9.5)	0.429
BP (mmHg)				
Systolic, mean (SD)	130.5 (14.1)	131.4 (14.2)	129.5 (14.2)	0.668
Diastolic, mean (SD)	75.7 (9.2)	77.0 (8.5)	74.2 (9.8)	0.322
Lipids (mmol/L)				
LDL, mean (SD)	2.37 (1.06)	2.3 (1.15)	2.40 (0.96)	0.844
HDL, mean (SD)	1.13 (0.30)	1.08 (0.30)	1.19 (0.30)	0.226
Ratio TC: HDL	3.47 (1.25)	3.41 (1.32)	3.54 (1.17)	0.745

*Significance testing for difference across sites

With respect to CVD medications, 44.7% of participants were taking two of the four classes of study medications, 40.4% were taking three and only 14.9% were on all four. Mean systolic (130.5 mmHg; SD 14.1) and diastolic (75.7 mmHg; SD 9.2) blood pressure readings were at targets for persons without kidney disease, which would be a systolic blood pressure of 130 mmHg and a diastolic blood pressure of 80 mmHg. As indicated by the standard deviations, there was some variation in both blood pressure readings, reflecting that some individual patients had blood pressure readings above these targets. Mean LDL cholesterol level at baseline was 2.37 [SD 1.06], which is slightly above the target level for persons with CVD, which is 2.0.

### Medication knowledge

**Fig. 3:**
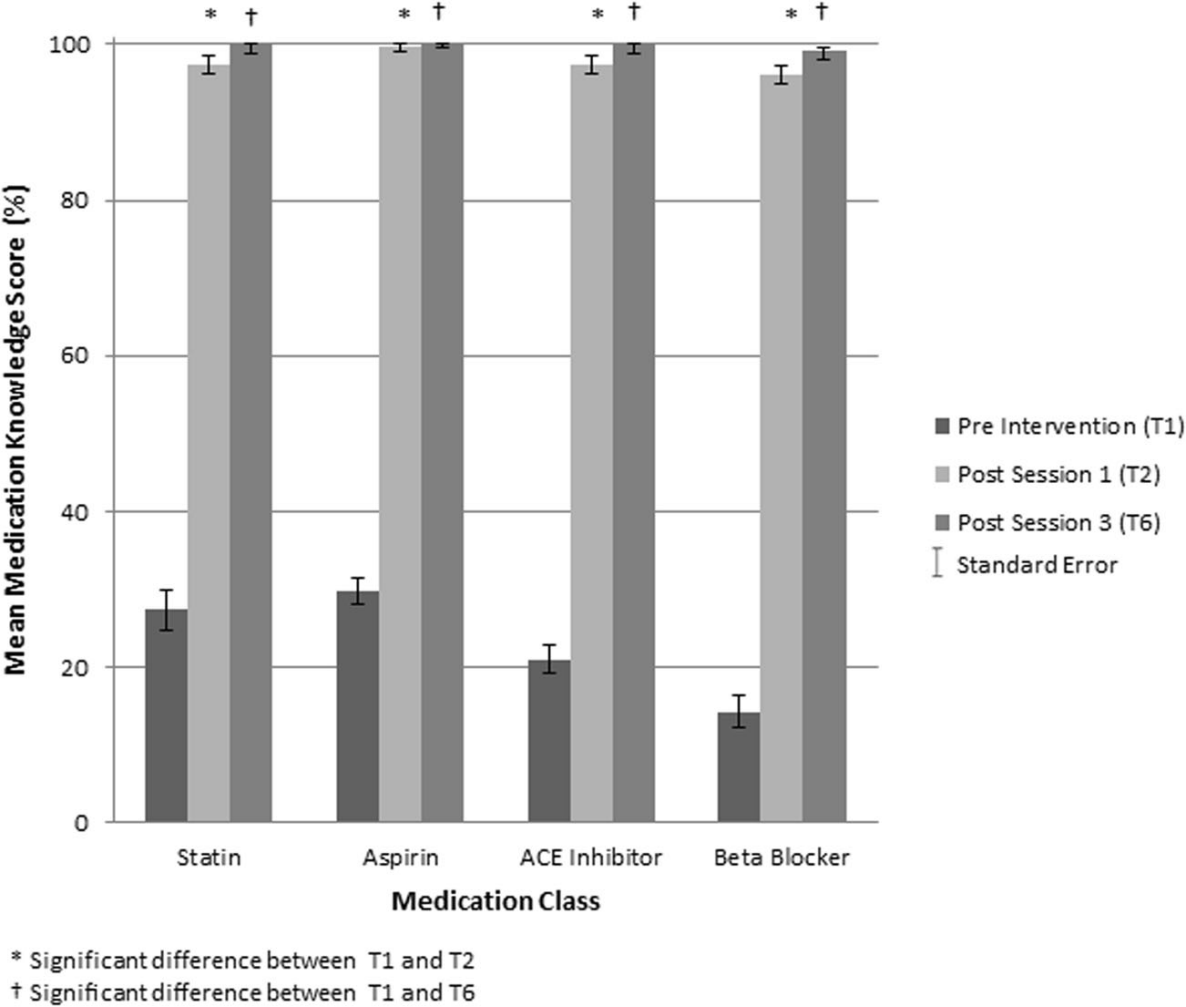
Unadjusted mean medication knowledge scores at T1, T2 and T6 for each medication class

Unadjusted mean medication knowledge scores were significantly higher following completion of the three education sessions compared to baseline across all four medication classes. The largest gains in knowledge scores occurred after the first educational session, with smaller additional gains following educational sessions 2 and 3 (Fig. 3). Adjusted GEE models (controlling for site and diabetes) showed mean knowledge scores post-intervention (T6) that were 3.3 to 6.1 times higher than baseline scores (T1) for the four included CVD medications (Table 3).

**Table 3:** Multivariable GEE models for CVD medications change in % items correct in knowledge questionnaire

	*n*	Pre knowledge (T1) score (mean [95%CI])	Post-knowledge (T6) score (mean [95%CI])	Exp(*B*) and 95%CI for Exp(*B*)	*p* value*
Statin					
Session 1	43	30.32 (26.28, 34.97)	98.24 (93.87, 100.00)	1.176 (1.02, 1.33)	< 0.001
Session 2	43	98.46 (96.86, 100.00)	99.46 (98.18, 100.00)	0.008 (0.00, 0.02)	0.076
Session 3	43	99.76 (99.30, 100.00)	100.00 (99.95, 100.00)	0.003 (0.00, 0.01)	0.312
Aspirin					
Session 1	35	29.94 (26.87, 33.37)	97.70 (94.24, 101.29)	1.183 (1.09, 1.28)	< 0.001
Session 2	34	99.10 (97.90, 100.00)	99.32 (98.31, 100.35)	0.002 (0.00, 0.01)	0.311
Session 3	34	100.00 (100.00, 100.00)	100.00 (100.00, 100.00)	1.000 (1.00, 1.00)	1.000
ACE inhibitor					
Session 1	29	20.77 (16.86, 25.58)	93.41 (82.89, 105.26)	1.504 (1.35, 1.65)	< 0.001
Session 2	29	95.46 (90.43, 100.77)	98.90 (96.08, 101.79)	0.035 (0.00, 0.07)	0.053
Session 3	29	99.35 (98.27, 100.44)	99.64 (99.03, 100.00)	0.003 (0.00, 0.01)	0.309
Beta blocker					
Session 1	21	16.32 (12.60, 21.13)	100.00 (90.77, 100.00)	1.817 (1.59, 2.06)	< 0.001
Session 2	21	94.94 (91.27, 98.75)	97.95 (96.05, 99.90)	0.031 (0.00, 0.06)	0.051
Session 3	21	97.20 (95.30, 99.14)	98.95 (97.57, 100.00)	0.018 (0.00, 0.03)	0.027

All models included site and diabetes comorbidity

*p value is the probability of the null hypothesis that Exp(*B*) = 1

### Health literacy practices

By the end of educational session 3, over 90% of participants were spontaneously (without prompting from the nurse) using the medication booklet and close to half of participants were spontaneously using their customized pill card to access information about their CVD medications (Table 4). There was a non-significant increase in participants who spontaneously access any source of medication information (pill bottle, pill card, medication booklet, own resource) post-intervention (T6) compared to baseline (T1).

**Table 4:** Spontaneous participant use of different CVD medication information sources

Information source	Pre-session 1 (T1), *n* (%)	Post-session 3 (T6), *n* (%)	*p* value*
Pill bottle	9 (19.15)	5 (10.64)	0.250
Pill card	0	21 (44.68)	< 0.001
Medication booklet	0	43 (91.49)	< 0.001
Own information	23 (48.94)	4 (8.51)	0.276
Any of the above	28 (59.57)	44 (93.62)	0.800

*Significance testing for difference between (T1) and (T6)

Participants were also significantly more likely to be answering questions from other people regarding their medications between sessions 2 and 3 (T5) compared to between sessions 1 and 2 (T3) (*p* = 0.018). There was a non-significant trend towards participants being more likely to ask their nurse or doctor questions about their medication at T5 compared to T3 (*p* = 0.0615).

## Discussion

We have demonstrated that our customized education program is highly effective in increasing medication knowledge among Indigenous peoples with CVD or at risk of CVD attending the program at an urban Indigenous health centre in Ontario, Canada. Mean medication knowledge scores at baseline were low enough to put participants at risk of medication error. Upon completion of our educational intervention, participants had near-perfect medication knowledge scores. Notably, almost all of the knowledge acquisition occurred after the first educational session, with very small mean knowledge score enhancements following sessions 2 and 3. In addition to these striking impacts on medication knowledge, our intervention was also effective in modifying participant health literacy behaviours. Following program completion, participants had adopted and were using the information resources provided to them as part of the program—91% were spontaneously referring to the program medication booklet and 45% were spontaneously using their customized pill card when seeking information about their medications. They were also more likely to answer other peoples’ questions about CVD medications.

The striking improvements in participant medication knowledge scores after a single 1 h structured educational session are remarkable, particularly given the context of lower levels of health literacy among our study population demonstrated in our pre-trial investigations (Downey et al. 2013). Importantly, participants also demonstrated health literacy behaviour changes, actively adapting the customized learning tools provided by the intervention (pill card and medication booklet) and answering other peoples’ questions regarding CVD medications. These positive results highlight the benefits of using an integrated systems level approach to health literacy that takes into account learning environment and processes, HCP–patient relationships, HCP health literacy teaching skills, style and content of information shared, and optimization of learning modalities and tools. At the same time, our findings challenge health literacy approaches that locate the problem as one of individual patient deficits.

Given the disproportionate burden of CVD morbidity and mortality experienced by Indigenous populations in Canada, our findings have implications for future research, practice and policy. First, it is disheartening to note that even though our inclusion criteria required patients to be taking a minimum of two of the four classes of CVD medications recommended for persons with CVD or at moderate risk of CVD, less than 15% of our study participants were on all four medication classes currently recommended for persons with or at moderate risk of CVD (Mancini et al. 2014; Tobe et al. 2011). This suggests the need for further research examining the quality of medical CVD management among Indigenous populations. Second, and on a more encouraging note, our study’s positive results with respect to improvements in medication knowledge and health literacy skills lay the groundwork for a larger, multi-site Canadian study, that includes longer term follow-up, and additional health literacy behaviour and clinical outcome measures. In addition, while the current intervention focused on CVD medications, the approach and program would be readily adaptable for other chronic diseases, including diabetes, that currently challenge Indigenous populations. Results of our international study, to be released shortly, support the generalizability of the findings of this Canadian site study across five diverse international Indigenous health service provider contexts.

Limitations of our study include its single arm design, small size, and relatively short follow-up period. As detailed elsewhere (Crengle et al. 2014), our relatively small study population and the risk of cross-participant contamination precluded a randomized design in this initial demonstration study. Final outcome measures were collected 4 to 8 weeks following the first educational session, and due to the high rates of knowledge uptake in this first session, there is some indication of retention of knowledge over time; however, longer term follow-up and the inclusion of clinical follow-up measures would be useful to better understand CVD health outcome impacts. We also note that the expansion of direct and observed health literacy assessment tools that has occurred since we designed this study provides opportunities to build in additional measures of health literacy assessment into future studies (Tobe et al. 2011). Finally, we note that a considerable number of potential participants could not be reached by phone or mail. Using point of care to recruit potential participants in future studies may be one way of overcoming this barrier.

Our protocol and processes are exemplary in the way that Indigenous leadership and community involvement were built into the study design. To our knowledge, our study is one of the first clinical trials to integrate benchmark Indigenous research leadership and partnership principles and protocols, including on-going leadership and participation of Indigenous community representatives on the study design and implementation team and an academic–community research, data-sharing and publication agreement that ensures community custodianship of study data and control of research publications.

## Conclusion

This customized health literacy education program was highly effective in increasing medication knowledge and health literacy practice among Indigenous people with CVD or at risk of CVD attending an urban Indigenous health centre in Canada. Within this context, we demonstrated that strategically addressing health literacy barriers at both the systems and individual health provider levels in a culturally relevant manner resulted in marked and rapid uptake of both CVD medication knowledge and health literacy practice in a population known to have lower levels of education and literacy compared to the general population. This challenges existing public health and health promotion models which commonly locate health literacy as a problem of Indigenous peoples which is pre-determined by existing inequities in levels of education and literacy in non-Indigenous languages and systems compared to the general Canadian population. Our findings are highly relevant to existing Indigenous health services and policy and signal the need for further testing and scale-up across diverse Indigenous contexts and other disease classes, including diabetes medications.
